# Dispositional gratitude, health-related factors, and lipid profiles in midlife: a biomarker study

**DOI:** 10.1038/s41598-022-09960-w

**Published:** 2022-04-11

**Authors:** Andree Hartanto, Nadyanna M. Majeed, Verity Y. Q. Lua, Joax Wong, Nicole R. Y. Chen

**Affiliations:** School of Social Sciences, Singapore Management University, 90 Stamford Road, Level 4, Singapore, 178903 Singapore

**Keywords:** Psychology, Medical research

## Abstract

Dispositional gratitude has emerged in the literature to be associated with many health benefits in measures ranging from self-reported health to biomarkers of cardiovascular risk. However, little is known about the link between dispositional gratitude and lipid profiles. Drawing from the Gratitude and Self-improvement Model that grateful individuals are more likely to strive for actual self-improvement such as engaging in healthy lifestyles, we investigated the relation between dispositional gratitude and serum lipid levels. Participants consisted of 1800 adults from the National Survey of Midlife Development in the United States (MIDUS) 2: Biomarker Project (*N* = 1054) and MIDUS Refresher: Biomarker Project (*N* = 746). Serum lipid profiles were measured through fasting blood samples. After controlling for demographics, use of antihyperlipidemic mediation, and personality traits, we found that higher dispositional gratitude was associated with lower triglyceride levels. Results also revealed that healthy diets and lower BMI partially mediated the gratitude-triglyceride association. However, some variations in the analytic method may influence the associations between gratitude and triglycerides levels. Our findings provide preliminary evidence suggesting dispositional gratitude as a promising psychological factor that is associated with a healthier lipid profile.

## Introduction

Unhealthy lipid profiles, such as high ratios of total cholesterol to high-density lipoprotein (HDL) cholesterol and high levels of triglycerides, are associated with an increased risk of cardiovascular disease such as coronary heart disease, peripheral arterial disease, and ischemic stroke^1–3^. Given the importance of healthy lipid profiles, a growing body of research has sought to investigate how psychological factors contribute to serum lipid levels. For example, Boehm and colleagues^4^ found that dispositional optimism was associated with higher HDL cholesterol and lower triglyceride levels. Meanwhile, four of the big five personality traits (i.e., lower conscientiousness, higher extraversion, lower emotional stability and lower openness to experience) were also found to be associated with elevated triglyceride levels and lower HDL cholesterol levels^5–7^. One promising psychological factor that has yet to be empirically investigated in relation to serum lipid levels is dispositional gratitude the dispositional tendency to perceive and appreciate the positives in life^8,9^.

Dispositional gratitude as a psychological factor has emerged in literature to be associated with a wide range of positive outcomes. Grateful people are those who appreciate the positive things in life^10^. Research has showed that those who are grateful are more likely to report higher life satisfaction^11,12^, experience positive affect^13,14^, adopt adaptive coping strategies^8,15^, and have fulfilling and positive relationships^16^. In addition to these psychosocial benefits, gratitude has been linked to benefits in physical well-being^17^. For instance, dispositional gratitude has been shown to predict higher self-rated health^17^, lower obesity^18^, and fewer physical health symptoms such as sleep disturbances, headaches, and gastrointestinal problems^19^. Dispositional gratitude has also been linked with better cardiovascular health as indexed by biomarkers such as tumor necrosis factor-alpha^20^, interleukin-6^21^ and hemoglobin A1c protein^22^. However, the link between dispositional gratitude and lipid profiles has remained unexplored.

Higher dispositional gratitude is expected to be associated with a healthier lipid profile because of its associations with health-related factors that can help in maintaining lipid levels. For instance, Hill and colleagues^17^ found that higher engagement in health activities in grateful older adults mediated the link between dispositional gratitude and self-rated health. Additionally, studies have found that gratitude reduces the risk of obesity by facilitating healthy eating behavior over time^23^ and predicts a significantly lower risk of alcohol and nicotine dependence^24^. These findings are consistent with the Gratitude and Self-Improvement Model^25^. According to the model, gratitude is an active emotion that motivates and energizes individual to exercise effort in executing a range of positive self-improvement behaviours. For example, grateful people are more likely to engage in positive reframing that encourage them to believe that they deserve positive outcomes for themselves and have self-efficacy of attaining them^26^. With the motivation to improve themselves, grateful people are more likely to strive for actual self-improvement such as engaging in healthy lifestyles, which may translate to healthier lipid profiles.

Taken together, the current study aimed to examine the association between dispositional gratitude and lipid profile markers indexed by the high-density lipoprotein (HDL) cholesterol levels, low-density lipoprotein (LDL) cholesterol levels and triglyceride levels. We hypothesized that dispositional gratitude would be positively associated with HDL cholesterol levels and negatively associated with LDL cholesterol levels and triglyceride levels, even after controlling for demographics^27–29^ as well as other well-established personality traits that have been found to correlate with dispositional gratitude and lipid profiles, such as dispositional optimism^30^ and the big five personality traits^31^. Lastly, we also aimed to explore how various lifestyle factors such as healthy eating, smoking, and alcohol intake contribute to the association between dispositional gratitude and lipid profiles. This would enable us to identify potential lifestyle-related pathways in the dispositional gratitude and lipid association. To test these hypotheses, four statistical models were estimated. In the first model, we estimated an unadjusted model with dispositional gratitude as the only predictor without entering any covariates. In the second model, we controlled for the use of hyperlipidemic agent medication^32^, and demographic variables of age, sex, education attainment, household income, and race^33^. In the third model, we controlled for dispositional traits that have previously been shown to influence lipid profile, which consisted of dispositional optimism^4^ and the big five personality traits^6,7^. The third model allowed us to test the incremental validity of dispositional gratitude and rule out the possibility that the hypothesized association between dispositional gratitude and lipid profile was simply due to its covariance with dispositional optimism and other well-established personality traits. In our final regression model, we additionally controlled for BMI, healthy eating, exercise, smoking, and alcohol intake (drinks per month) to explore whether BMI and health behaviors were possible pathways between dispositional gratitude and lipid profiles.

## Methods

### Participants

The current study consisted of 1800 adults (see Table 1) from the National Survey of Midlife Development in the United States (MIDUS) 2: Biomarker Project (*N* = 1054) and MIDUS Refresher: Biomarker Project (*N* = 746), ranging in age from 26 to 86 years old. Most participants were aged between 40 and 65 (64.94%), while 10.00% of participants were aged below 40 and 25.06% of participants were above 65 years of age. In terms of ethnic background, 1578 participants (88.06%) identified themselves as White. The MIDUS 2: Biomarker Project^34^, conducted from 2004 to 2009, is a subset of a national probability sample of 7108 English-speaking non-institutionalized adults, aged 35–86, recruited through random sampling across the United States. The MIDUS Refresher: Biomarker Project^35^, conducted from 2012 to 2016, is a younger distinct cohort and a subset of the MIDUS Refresher baseline cohort comprising a national probability sample of 3577 adults aged 25–74.

**Table 1:** Demographics, health behaviors, lipid profile, and other main characteristics of participants in the biomarker projects.

Variables	*N*	*M* (*SD*)	Range
**Demographics**			
Age at biomarker assessment (years)	1800	56.17 (12.69)	26–86
Sex (% male)	1800	47.28%	
Race (% White)	1792	88.06%	
Education^a^	1796	8.02 (2.43)	1–12
Household income (in thousands)	1752	82.38 (63.15)	0–300
**Personal dispositions**			
Dispositional gratitude	1797	6.25 (0.82)	1.50–7.00
Dispositional optimism	1792	23.52 (4.82)	6–30
Agreeableness	1794	3.39 (0.52)	1.20–4.00
Conscientiousness	1794	3.38 (0.47)	1.40–4.00
Extraversion	1794	3.11 (0.58)	1.20–4.00
Emotional stability	1794	2.05 (0.64)	1.00–4.00
Openness to experience	1789	2.98 (0.52)	1.00–4.00
**Health-related**			
Alcohol consumption (drinks per month)	1800	16.05 (27.84)	0–360
Body mass index	1799	29.51 (6.64)	14.99–77.58
Exercise (% exercise regularly)	1800	78.00%	
Healthy eating index	1794	16.99 (2.69)	8–24
Smoking (% current smoker)	1799	10.23%	
Antihyperlipidemic agent medication (% on medication)	1800	29.67%	
**Lipid profile**			
Triglycerides (mg/DL)	1785	128.37 (117.75)	25–3299
Total cholesterol (mg/dL)	1785	184.86 (39.20)	70–439
High-density lipoprotein (HDL) cholesterol (mg/dL)	1782	56.51 (18.68)	19–137
Low-density lipoprotein (LDL) cholesterol (mg/dL)	1782	102.87 (34.42)	2.80–283.00

Values are shown before imputation.

^a^Education attainment was rated on a scale of 1 (*No school*) to 12 *(PhD, EdD, MD, LLB, LLD, JD, or other professional degree*).

Both MIDUS 2: Biomarker Project and MIDUS Refresher: Biomarker Project utilized the same data collection methodology and employed identical measures. Participants in both projects were invited to visit one of the three clinical research centers (University of Wisconsin-Madison; University of California, Los Angeles; and Georgetown University) for an overnight hospital stay for an in-depth health assessment that included a lipid panel of total cholesterol, LDL, HDL, and triglycerides through the collection of a fasting blood sample before breakfast on the second morning of the hospital visit. Data collection at the University of Wisconsin-Madison, University of California, Los Angeles, and Georgetown University for both studies was approved by the Health Sciences IRBs at the University of Wisconsin-Madison and was conducted accordance to approved guidelines and regulations. All participants provided written informed consent prior to participation. The secondary analysis of both MIDUS 2 and MIDUS Refresher datasets was approved by the IRB at the Singapore Management University.

## Measures

### Personal dispositions

#### Dispositional gratitude

Dispositional gratitude was measured using a two-item shortened version of the Gratitude Questionnaire^10^. Participants rated their agreement with the statements “I am grateful to a wide variety of people” and “I have so much in life to be thankful for” on a 7-point scale (1 = *Strongly disagree*, 7 = *Strongly agree*). The mean score across both items was computed with higher scores indicating higher dispositional gratitude. The shortened version of the gratitude scale had good reliability in the current study (*α* = 0.71) and has been widely shown in previous studies in predicting theoretically relevant constructs^36,37^.

#### Dispositional optimism

Dispositional optimism was measured using the six-item Life Orientation Test^38^ without filler items. Participants rated themselves on a 5-point scale (1 = *A lot agree*, 5 = *A lot disagree*) on three items measuring optimism (e.g., “In uncertain times, I usually expect the best.”) and three items measuring pessimism (e.g., “If something can go wrong for me, it will.”). Scores on the pessimism items were reverse coded and dispositional optimism was measured as the sum of scores across all six items (*α* = 0.83). Higher scores indicated greater dispositional optimism.

#### Five-factor personality traits

The five-factor personality traits were assessed using a 4-point scale (1 = *A lot*, 4 = *Not at all*) on a series of self-descriptive adjectives which was developed for use in the MIDUS studies by combining a set of existing personality inventories, and validated in a study consisting of 1000 participants^39^. Agreeableness (e.g., helpful, warm, caring, softhearted, and sympathetic; *α* = 0.81), conscientiousness (e.g., organized, responsible, hardworking, careless, and thorough; *α* = 0.70), and extraversion (e.g., outgoing, friendly, lively, active, and talkative; *α* = 0.78) were measured via five adjectives each. Emotional stability was measured using four adjectives (e.g., moody, worrying, nervous, calm; *α* = 0.75), while openness to experience was measured using seven adjectives (e.g., curious, imaginative, intelligent, curious, broad-minded, sophisticated, and adventurous; *α* = 0.76). All items were scored such that higher scores indicated higher standings in that particular dimension, except for emotional stability which was scored such that lower scores indicated more emotional stability.

### Demographics

#### Education attainment

Education attainment was measured by asking the participants “what is the highest grade of school or year of college you completed?” through a phone interview. It was rated on a 12-points scale (1 = No school/some grade school (1–6); 2 = Eight grade/junior high school (7–8); 3 = Some high school (9–12 no diploma/no GED); 4 = GED; 5 = Graduated from high school; 6 = 1–2 years of college, no degree yet; 7 = 3 or more years of college, no degree yet; 8 = Graduated from a 2 year college or vocational school, or associate’s degree; 9 = Graduated from a 4 or 5 year college, or bachelor’s degree; 10 = Some graduate school; 11 = Master’s degree; 12 = Ph.D., ED.D., MD, DDS, LLB, LLD, JD, or other professional degree).

### Health-related variables

#### Serum lipid profile

Participants’ fasting blood samples were obtained at one of the three clinical research sites on the second morning of their 2 day visit. The samples were stored in a − 80 to − 60 °C freezer before the frozen serums were transported on dry ice to Meriter Laboratories (Madison, Wisconsin), where the serums were then stored at − 65 °C. All assays were performed with a Cobas Integra analyzer (Roche Diagnostics, Indianapolis, Indiana).

Total cholesterol, HDL cholesterol, and triglyceride levels were determined using enzymatic colorimetric assays in MIDUS 2 and in MIDUS Refresher. The inter-assay and intra-assay coefficients of variability for total cholesterol were 2.65% and 0.51–0.81% respectively in MIDUS 2, and 4.13% and 0.51–0.81% respectively in MIDUS Refresher. For HDL cholesterol, the inter-assay and intra-assay coefficients of variability were 1.01% and 1.6% in MIDUS 2 and 2.51% and 1.6% in MIDUS Refresher respectively. Finally, the inter-assay and intra-assay coefficients of variability for triglyceride levels were 6.52% and 1.1–1.4% in MIDUS 2, and 3.56% and 1.1–1.4% in MIDUS Refresher respectively. Low-density lipoprotein cholesterol was estimated using the Friedewald formula^40^. Triglyceride levels above 400 mg/dl were replaced with 400 mg/dl to calculate LDL cholesterol levels. The inter-assay coefficients of variability for LDL cholesterol was 10.11% in MIDUS 2 and 4.7% in MIDUS Refresher.

#### Use of antihyperlipidemic agent medication

The use of cholesterol medication was recorded by requiring participants to bring all their medication in their original containers during the study to ensure accuracy. Each medication was matched through the Lexicomp® Lexi-Data database to their generic names and drug IDs, and ultimately to their therapeutic and pharmacologic class codes. The use of any form of antihyperlipidemic agent medication (e.g., HMG-CoA reductase inhibitor, fibric acid derivatives, bile acid sequestrants, cholesterol absorption inhibitors) was dummy coded (1 = *yes*, 0 = *no*).

#### Healthy eating index

A Healthy Eating Index^41^ based on McCullough & Willett’s Alternative Healthy Eating Index (AHEI; 2006) was computed to measure participants’ adherence to the Dietary Guidelines for Americans and the Food Guide Pyramid. Based on the measures used in the MIDUS 2 and MIDUS Refresher biomarker projects, five items (e.g., “On an average day, how many sugared beverages do you drink (e.g. soda, sports drinks, bottled drinks, fruit drinks)?”) covering six of the 11 components in the AHEI were used to compute participants’ Healthy Eating Index scores^41^. Specifically, the Healthy Eating Index considered participants’ intake of sugar-sweetened beverages (reverse coded), vegetables, fruit, non-meat protein, beef and high fat meat (reverse coded), and fish. Participants rated each item on a 5-point scale, depending on the frequency in which they engaged in the behavior. The scores of each item were summed to obtain a Healthy Eating Index score, whereby a higher score indicated healthier eating behaviors.

### Analytic plan

The goal of the current study was to examine the association between dispositional gratitude and lipid profiles indexed by three well-established indicators: HDL cholesterol levels, LDL cholesterol levels, and triglyceride levels. To account for the small amount of missing data (0.44% missing in total across all variables), 10 complete datasets were created through Markov Chain Monte Carlo (MCMC) multiple imputation. All outcome indices were winsorized to 3 *SD*s to reduce the influence of extreme outliers. In our main analyses, linear regressions were conducted with dispositional gratitude as a predictor of each of the three lipid profile measures. In follow-up analyses, linear (for continuous outcomes) and logistic (for binary outcomes) regressions were conducted to examine gratitude as a predictor of alcohol consumption, BMI, exercise, healthy eating, and smoking. Additionally, serial mediation analyses were also conducted to examine possible mechanisms behind the gratitude-lipid profile association.

### Transparency, openness, and data availability

All MIDUS datasets and documentation are archived and publicly available at the ICPSR repository (http://www.icpsr.umich.edu/) at the University of Michigan. The analytic code used in the current work has been made publicly available on Researchbox (#142: https://researchbox.org/142). All analyses were conducted in R version 3.6.3^42^. Descriptive statistics and scale reliabilities were computed via psych version 2.1.6^43^. MCMC imputation (via a fully conditional specification procedure) and pooling of analyses on the imputed datasets were carried out using mice version 3.13.0^44^ and mitml version 0.4-3^45^. Standardized coefficients were obtained by running the analyses on a standardized version of the dataset created by effectsize version 0.4.5^46^. Serial mediation analysis was performed by PROCESS version 4.0.1 for R^47^.

## Results

### Main analyses

#### HDL levels

Dispositional gratitude significantly predicted HDL levels in Model 1 (β = 0.08, *b* = 1.80, *SE* = 0.53, 95% CI = [0.77, 2.82], *p* < 0.001), such that individuals higher in gratitude exhibited higher levels of HDL in their blood as compared to individuals lower in gratitude. However, the association between gratitude and HDL levels became non-significant in Model 2 (β = 0.01, *b* = 0.31, *SE* = 0.49, 95% CI = [− 0.65, 1.27], *p* = 0.525), suggesting that the link between dispositional gratitude and HDL can be accounted for by medication use and demographic factors. The results were consistent after additionally controlling for personality factors in Model 3 (β = 0.00, *b* = 0.02, *SE* = 0.53, 95% CI = [− 1.01, 1.05], *p* = 0.973) and health-related factors of smoking, alcohol consumption, BMI, healthy eating, and exercise in Model 4 (β = − 0.01, *b* = − 0.18, *SE* = 0.47, 95% CI = [− 1.10, 0.75], *p* = 0.706; see Table 2).

**Table 2:** Model summaries with HDL as the outcome variable.

Predictor	Model 1				Model 2				Model 3				Model 4			
	β	*b*	*SE*	*p*	β	*b*	*SE*	*p*	β	*b*	*SE*	*p*	β	*b*	*SE*	*p*
Dispositional gratitude	.08	1.80	0.52	** < .001**	.01	0.31	0.49	.525	.00	0.02	0.53	.973	− .01	− 0.18	0.47	.706
Antihyperlipidemic medication use (0 = *no*, 1 = *yes*)					− .21	− 3.80	0.93	** < .001**	− .21	− 3.79	0.93	** < .001**	− .10	− 1.74	0.84	**.039**
**Demographics**																
Age					.13	0.19	0.03	** < .001**	.13	0.18	0.03	** < .001**	.08	0.11	0.03	** < .001**
Sex (0 = *female*, 1 = *male*)					− .75	− 13.71	0.81	** < .001**	− .77	− 14.13	0.85	** < .001**	− .80	− 14.67	0.79	** < .001**
Race (0 = *White*, 1 = *non-White*)					.09	1.57	1.24	.205	.08	1.50	1.24	.223	.15	2.80	1.11	**.012**
Education					.11	0.84	0.17	** < .001**	.09	0.69	0.18	** < .001**	.03	0.22	0.16	.182
Household income					.04	0.01	0.01	.068	.03	0.01	0.01	.193	.00	0.00	0.01	.957
**Personality**																
Dispositional optimism									.08	0.30	0.11	**.004**	.05	0.20	0.10	**.038**
Agreeableness									− .10	− 3.64	0.95	** < .001**	− .03	− 1.21	0.86	.162
Conscientiousness									.04	1.56	0.92	.090	.02	0.83	0.83	.316
Extraversion									.05	1.59	0.88	.070	− .01	− 0.17	0.79	.827
Emotional stability									.03	0.90	0.72	.214	.01	0.37	0.65	.573
Openness to experience									.03	0.91	0.94	.331	.02	0.62	0.85	.462
**Health-related**																
Alcohol consumption													.26	0.17	0.01	** < .001**
BMI													− .27	− 0.73	0.06	** < .001**
Exercise (0 = *do not exercise regularly*, 1 = *exercise regularly*)													.12	2.25	0.88	**.010**
Healthy Eating Index													.10	0.65	0.14	** < .001**
Smoking (0 = *non-smoker*, 1 = *smoker*)													− .31	− 5.61	1.22	** < .001**

*N* = 1800. β = standardized slope coefficient, *b* = unstandardized slope coefficient, *SE* = standard error of the slope coefficient. Bolded *p*-values indicate statistical significance at the .05 level.

#### LDL levels

Dispositional gratitude did not significantly predict LDL levels in Model 1 (β = − 0.00, *b* = − 0.19, *SE* = 0.97, 95% CI = [− 2.09, 1.72], *p* = 0.847). This pattern was consistent even after controlling for medication and demographic factors in Model 2 (β = − 0.02, *b* = − 0.74, *SE* = 0.93, 95% CI = [− 2.55, 1.08], *p* = 0.425), personality factors in Model 3 (β = − 0.02, *b* = − 0.74, *SE* = 1.01, 95% CI = [− 2.72, 1.23], *p* = 0.460), and health-related factors in Model 4 (β = − 0.01, *b* = − 0.35, *SE* = 1.01, 95% CI = [− 2.32, 1.62], *p* = 0.729; see Table 3).

**Table 3:** Model summaries with LDL as the outcome variable.

Predictor	Model 1				Model 2				Model 3				Model 4			
	β	*b*	*SE*	*p*	β	*b*	*SE*	*p*	β	*b*	*SE*	*p*	β	*b*	*SE*	*p*
Dispositional gratitude	− .00	− 0.19	0.97	.847	− .02	− 0.74	0.93	.425	− .02	− 0.74	1.01	.460	− .01	-0.35	1.01	.729
Antihyperlipidemic medication use (0 = *no*, 1 = *yes*)					− .77	− 25.92	1.77	** < .001**	− .77	− 25.80	1.77	** < .001**	− .78	− 26.32	1.78	** < .001**
**Demographics**																
Age					.02	0.05	0.06	.394	.02	0.05	0.07	.445	.04	0.10	0.07	.152
Sex (0 = *female*, 1 = *male*)					.01	0.36	1.54	.815	.01	0.27	1.63	.867	− .02	− 0.65	1.69	.702
Race (0 = *White*, 1 = *non-White*)					− .17	− 5.61	2.34	**.016**	− .17	− 5.87	2.35	** < .001**	− .19	− 6.41	2.34	**.006**
Education					− .08	− 1.17	0.33	** < .001**	− .09	− 1.22	0.34	** < .001**	− .06	− 0.86	0.35	**.013**
Household income					.00	0.00	0.01	.908	.00	0.00	0.01	.864	.01	0.01	0.01	.591
**Personality**																
Dispositional optimism									− .02	− 0.15	0.20	.471	− .01	− 0.06	0.20	.763
Agreeableness									.03	2.17	1.82	.234	.02	1.19	1.83	.515
Conscientiousness									− .03	− 2.30	1.76	.191	− .02	− 1.71	1.77	.332
Extraversion									− .02	− 0.98	1.67	.556	− .01	− 0.69	1.67	.681
Emotional stability									− .01	− 0.76	1.39	.582	− .02	− 0.97	1.39	.483
Openness to experience									.04	2.71	1.76	.122	.04	2.82	1.76	.110
**Health-related**																
Alcohol consumption													− .02	− 0.02	0.03	.477
BMI													.05	0.26	0.12	**.029**
Exercise (0 = *do not exercise regularly*, 1 = *exercise regularly*)													.00	0.07	1.89	.972
Healthy Eating Index													− .06	− 0.80	0.30	**.008**
Smoking (0 = *non-smoker*, 1 = *smoker*)													.24	7.92	2.61	**.002**

*N* = 1800. β = standardized slope coefficient, *b* = unstandardized slope coefficient, *SE* = standard error of the slope coefficient. Bolded *p*-values indicate statistical significance at the .05 level.

#### Triglyceride levels

Dispositional gratitude significantly predicted triglyceride levels in Model 1 (β = − 0.10, *b* = − 8.98, *SE* = 2.11, 95% CI = [− 13.11, − 4.85], *p* < 0.001), such that individuals higher in gratitude exhibited lower levels of triglycerides in their blood as compared to individuals lower in gratitude. This pattern was consistent even after controlling for medication and demographic factors in Model 2 (β = − 0.06, *b* = − 5.65, *SE* = 2.10, 95% CI = [− 9.77, − 1.54], *p* = 0.007), and after additionally controlling for personality factors in Model 3 (β =−0.06, *b* =+5.05, *SE* = 2.29, 95% CI = [− 9.54, − 0.56], *p* = 0.028). However, the association between gratitude and triglyceride levels became non-significant after including smoking, alcohol consumption, BMI, healthy eating, and exercise as covariates in Model 4 (β =−0.04, *b* = − 3.20, *SE* = 2.21, 95% CI = [− 7.54, 1.14], *p* = 0.148; see Table 4), suggesting the link between dispositional gratitude and triglycerides can be accounted for by health-related factors.

**Table 4:** Model summaries with triglycerides as the outcome variable.

Predictor	Model 1				Model 2				Model 3				Model 4			
	β	*b*	*SE*	*p*	β	*b*	*SE*	*p*	β	*b*	*SE*	*p*	β	*b*	*SE*	*p*
Dispositional gratitude	− 0.10	− 8.98	2.11	** < .001**	− 0.06	− 5.65	2.10	**.007**	− 0.06	− 5.05	2.29	**.028**	− 0.04	− 3.20	2.21	.148
Antihyperlipidemic medication use (0 = *no*, 1 = *yes*)					0.15	10.72	3.96	**.007**	0.15	10.65	3.94	**.007**	0.05	3.99	3.82	.297
**Demographics**																
Age					− 0.06	− 0.33	0.15	**.025**	− 0.04	− 0.23	0.15	.119	− 0.01	− 0.04	0.14	.760
Sex (0 = *female*, 1 = *male*)					0.38	27.52	3.44	** < .001**	0.41	29.56	3.61	** < .001**	0.34	24.80	3.60	** < .001**
Race (0 = *White*, 1 = *non-White*)					− 0.22	− 15.71	5.29	**.003**	− 0.23	− 16.53	5.28	**.002**	− 0.30	− 21.85	5.12	** < .001**
Education					− 0.07	− 2.17	0.74	**.003**	− 0.06	− 1.69	0.77	**.028**	− 0.00	− 0.11	0.75	.886
Household income					− 0.08	− 0.09	0.03	**.002**	− 0.07	− 0.08	0.03	**.007**	− 0.06	− 0.07	0.03	**.021**
**Personality**																
Dispositional optimism									− 0.07	− 1.08	0.45	**.017**	− 0.05	− 0.75	0.44	.083
Agreeableness									0.06	8.43	4.05	**.037**	0.01	2.03	3.91	.605
Conscientiousness									− 0.03	− 5.31	3.91	.174	− 0.00	− 0.48	3.76	.899
Extraversion									0.04	5.09	3.74	.173	0.07	8.54	3.59	**.017**
Emotional stability									0.05	5.92	3.08	.054	0.06	6.24	2.97	**.036**
Openness to experience									0.02	3.05	3.96	.440	0.02	2.95	3.81	.438
**Health-related**																
Alcohol consumption													0.01	0.02	0.06	.755
BMI													0.25	2.76	0.27	** < .001**
Exercise (0 = *do not exercise regularly*, 1 = *exercise regularly*)													− 0.18	− 13.12	4.06	**.001**
Healthy Eating Index													− 0.09	− 2.39	0.65	** < .001**
Smoking (0 = *non-smoker*, 1 = *smoker*)													0.13	9.34	5.71	.102

*N* = 1800. β = standardized slope coefficient, *b* = unstandardized slope coefficient, *SE* = standard error of the slope coefficient. Bolded *p*-values indicate statistical significance at the .05 level.

### Sensitivity analyses

Following up on the significant associations between dispositional gratitude and triglycerides in Model 1, Model 2, and Model 3, sensitivity analyses were performed to examine if the current findings remained robust when we varied our method of analysis, such as using other methods to handle missing data, other data pre-processing choices, analyzing the MIDUS 2 and MIDUS Refresher datasets separately, or applying adjustments for multiple comparisons (see Table 5). We found that the original results were generally robust across the various analytic choices for Model 1 (unadjusted) and Model 2 (controlling for medication use and demographics), though results were inconsistent in terms of statistical significance in Model 3 (controlling for personality). However, it should be noted that the predictive strength of gratitude itself was quite consistent from Model 2 (|β|s = [0.05, 0.08]) to Model 3 (|β|s = [0.05, 0.07]) (see Supplementary Material for more sensitivity analyses).

**Table 5:** Coefficients of gratitude predicting triglyceride levels in sensitivity analyses.

Variation in analysis	Model 1		Model 2		Model 3	
	β	*p*	β	*p*	β	*p*
Original results (for comparison)	− .10	** < .001**	− .06	**.007**	− .06	**.028**
**Alternative data pre-processing choices**						
Gratitude reflected and log-transformed	.10^a^	** < .001**	.06^a^	**.011**	.05^a^	**.033**
Triglyceride level log-transformed (after winsorization at 3 *SD*)	− .10	** < .001**	− .06	**.013**	− .05	.058
Winsorization (3 *SD*) applied to all variables	− .11	** < .001**	− .07	**.004**	− .06	**.017**
Winsorization (4 *SD*) applied to all variables	− .10	** < .001**	− .07	**.004**	− .07	**.013**
**Sub-sample analyses**						
Only participants with complete data (i.e., listwise deletion; *n* = 1704)	− .11	** < .001**	− .07	**.003**	− .07	**.010**
Only participants not taking antihyperlipidemic medication (*n* = 1266)	− .11	** < .001**	− .07	**.022**	− .05	.089
Only MIDUS 2 data (*n* = 1054)	− .09	**.002**	− .05	.124	− .05	.175
Only MIDUS Refresher data (*n* = 746)	− .12	**.002**	− .08	**.022**	− .06	.130
**Adjustment for multiple comparisons**^**b**^						
Adjustment with Hommel procedure	− .10	**< .001**	− .06	**.050**	− .06	.168
Adjustment with Bonferroni procedure	− .10	**< .001**	− .06	.064	− .06	.252
Adjustment with Benjamini–Hochberg procedure	− .10	**< .001**	− .06	**.021**	− .06	.063

Bolded *p*-values indicate statistical significance at the .05 level.

^a^Coefficients were opposite in sign compared to the rest of the results because gratitude was reflected (i.e., reversed).

^b^*p*-value adjustments were conducted taking into account all three dependent variables (triglycerides, LDL, HDL) across the first three models (i.e., a total of nine *p*-values).

### Exploratory serial mediation

Following up on the results of Model 4 with triglycerides as the lipid index of interest, dispositional gratitude was consistently associated with lower levels of alcohol consumption, the higher scores on the Healthy Eating Index, and lower probability of being a smoker (see Table 6). Of these three health-related factors, only the Healthy Eating Index was a unique significant predictor of serum triglyceride levels (see Model 4 in Table 4). In addition, BMI and exercise were also significant predictors of serum triglyceride levels (see Model 4 in Table 4).

**Table 6:** Gratitude as a predictor of alcohol consumption, BMI, exercise, healthy eating index, and smoking.

Model	Alcohol consumption^a^		BMI^a^		Exercise^b^		Healthy eating index^a^		Smoking^b^	
	β	*p*	β	*p*	β	*p*	β	*p*	β	*p*
Model 1	− .08	** < .001**	− .07	**.002**	.15	**.007**	.14	** < .001**	− .29	** < .001**
Model 2	− .06	**.007**	− .04	.055	.12	**.035**	.09	** < .001**	− .23	**.001**
Model 3	− .06	**.024**	− .04	.157	.09	.163	.07	**.003**	− .17	**.027**

*N* = 1800. β = standardized slope coefficient.

Bolded *p* values indicate significance at the .05 level. Model 1 was an unadjusted model with dispositional gratitude as the only predictor without entering any other covariates. Model 2 additionally controlled for age, sex, education attainment, household income, race, and the use of hyperlipidemic agent medication. Model 3 additionally controlled for dispositional optimism, agreeableness, conscientiousness, extraversion, emotional stability, and openness to experience.

^a^Linear regression. ^b^Logistic regression.

**Figure 1:**
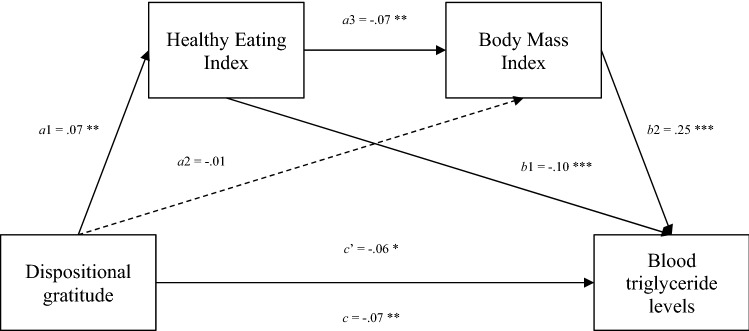
Serial mediation model for triglyceride levels. *Note* N = 1704. Serial mediation modelling demonstrates the influence of dispositional gratitude on blood triglyceride levels via the healthy eating index and body mass Index. a1, a2, a3, b1, b2, c, and c’ represent path coefficients in standardized forms. c and c’ represent the total effect and direct effect respectively. The model additionally controlled for age, sex, education attainment, household income, race, the use of hyperlipidemic agent medication, dispositional optimism, agreeableness, conscientiousness, extraversion, emotional stability, and openness to experience. Listwise deletion was used as an imputation method for the serial mediation analysis. Listwise deletion was used as Markov Chain Monte Carlo (MCMC) multiple imputation using mice version 3.13.0 and mitml version 0.4-3 was not compatible with PROCESS version 4.0.1 for R.

Hence, to further probe the underlying mechanism, an exploratory serial mediation analysis was conducted with lipid profile measured by serum triglyceride levels as the final outcome variable (Y), dispositional gratitude as the predictor variable (X), and health-related factors of healthy eating (M1) and BMI (M2) as the serial mediators, while controlling for medication, demographics, and personality covariates. As shown in the Fig. 1, the total effect (*c*) of gratitude on blood triglyceride levels was significant, β =−0.07, *b* = − 6.28, *SE* = 2.38, 95% CI = [− 10.95, − 1.62], *p* = 0.008. The bias-corrected bootstrap resampling method (5000 samples) showed a significant indirect effect of gratitude on blood triglyceride levels via the Healthy Eating Index and Body Mass Index (dispositional gratitude → healthy eating → BMI → triglyceride levels), β = − 0.001, *b* =−0.11, *SE* = 0.06, 95% CI = [− 0.24, − 0.02]. The residual direct effect (*c*’) of gratitude on blood triglyceride levels was also significant (β = − 0.06, *b* = − 5.25, *SE* = 2.30, 95% CI = [− 9.75, − 0.74], *p* = 0.022), suggesting only partial mediation via the serial healthy eating and BMI pathway.

## Discussion

Using a large national sample of midlife adults in the United States, the current study aimed to examine the unique link between dispositional gratitude and lipid profiles. Overall, we did not find any consistent evidence that dispositional gratitude was associated with LDL and HDL cholesterol levels. However, the current work suggests that individuals who were higher in dispositional gratitude displayed lower levels of triglyceride, even after controlling for demographic covariates. More importantly, the finding remained significant when other well-established psychological factors, such as trait optimism and the big five personality traits, were controlled for in the model, suggesting that the link between dispositional gratitude and triglyceride levels was above and beyond the influence of these well-established psychological factors. This result may support the incremental validity of dispositional gratitude in predicting some aspect of healthy lipid profiles.

Our analyses also identified a behavioral pathway that may serve as a mechanism underlying the healthy lipid profiles in individuals with dispositional gratitude. Specifically, healthier diets which contributed to lower BMI in individuals with dispositional gratitude partially mediated the link between dispositional gratitude and serum triglyceride levels. While healthy eating and regular exercise both predicted lower levels of serum triglyceride levels, dispositional gratitude was only uniquely associated with healthy eating but not regular exercise. The current finding is consistent with a past study which found that increasing state gratitude promoted healthy eating behaviors over time^23^. Our finding is also consistent with the Gratitude and Self-improvement Model^25^ which argues that gratitude as an active emotion that motivates and energizes one to exercise effort in engaging in self-improvement behaviors.

Despite the positive findings related to the associations between dispositional gratitude and triglycerides levels, we noted that the effect sizes of the associations were small. More importantly, some variations of the analysis method may influence the statistical significance of the associations between gratitude and triglycerides levels after controlling for trait optimism and the big five personality traits. While our sensitivity analyses in Model 1 (unadjusted) and Model 2 (controlling for medication use and demographics) showed that the associations were robust, the association between dispositional gratitude and triglycerides levels in Model 3 (controlling for personality) was not significant once participants not taking antihyperlipidemic medication were excluded or adjustment for multiple comparisons were conducted. The findings from our sensitivity analyses in Model 3 may suggest that the link between dispositional gratitude and triglyceride levels may not be unique and could be driven by its covariance with other well-established personality traits that are linked with lipid profiles, such as dispositional optimism^4^. Due to the small effect size, it is also important for futures studies to replicate the current finding to examine the robustness of the association between dispositional gratitude and triglyceride levels.

Several limitations of the current study were noteworthy. For instance, the finding of the current study was mainly based on a cross-sectional design, which limits conclusions on causality. Although we have systematically controlled for possible confounding variables, such as demographics, antihyperlipidemic medication use, dispositional optimism, and the big five personality traits, unaccounted-for third variables could still confound the current results. Furthermore, reverse causation is plausible, where healthier lipid profiles may contribute to higher dispositional gratitude. As gratitude has been shown to be modifiable even with a short intervention^48–50^, the current findings should be followed up by a high-powered gratitude intervention study in the future to provide causal evidence. Moreover, future studies should replicate the current finding in other cultures and age cohorts to ascertain generalizability, as our sample was limited to midlife adults in the United States.

Taken together, with these limitations in mind, the current study highlighted dispositional gratitude as a promising and unique psychological factor that is associated with a healthier lipid profile. However, it is noteworthy that the effect size of the association between dispositional gratitude and cholesterol levels was small. Our study contributes to the understanding on how perceiving and appreciating the positives in life could be associated with a reduction in the risk of cardiovascular disease and an improvement in physical health.

## Supplementary Information

Supplementary Information.

## Data Availability

All MIDUS datasets and documentation are archived and publicly available at the ICPSR repository (http://www.icpsr.umich.edu/) at the University of Michigan. The analytic code used in the current work has been made publicly available on Researchbox (#142: https://researchbox.org/142).
