# Supplementary material for: Dispositional gratitude, health-related factors, and lipid profiles in midlife: a biomarker study
Source: Sci Rep. 2022 Apr 11;12:6034. doi: 10.1038/s41598-022-09960-w (PMC9001645; doi:10.1038/s41598-022-09960-w)
Supplement: Supplementary file 1 — Supplementary Information. [file 41598_2022_9960_MOESM1_ESM.docx]

**Table A**

*Coefficients of Gratitude Predicting HDL Cholesterol Levels in Sensitivity Analyses*

| Variation in analysis | Model 1 | |  | Model 2 | |  | Model 3 | |
| --- | --- | --- | --- | --- | --- | --- | --- | --- |
|  | β | *p* |  | β | *p* |  | β | *p* |
| **Original results (for comparison)** | .08 | **< .001** |  | .01 | .525 |  | .00 | .973 |
| **Alternative data pre-processing choices** |  |  |  |  |  |  |  |  |
| Gratitude reflected and log-transformed | -.08 | **.001** |  | -.01 | .776 |  | .01 | .713 |
| HDL level log-transformed (after winsorization at 3 *SD*) | .09 | **< .001** |  | .02 | .364 |  | .00 | .885 |
| Winsorization (3 *SD*) applied to all variables | .08 | **< .001** |  | .01 | .522 |  | .00 | .993 |
| Winsorization (4 *SD*) applied to all variables | .08 | **< .001** |  | .01 | .517 |  | .00 | .933 |
| **Sub-sample analyses** |  |  |  |  |  |  |  |  |
| Only participants with complete data (i.e., listwise deletion; *n* = 1704) | .09 | **<.001** |  | .03 | .253 |  | .01 | .594 |
| Only participants not taking antihyperlipidemic medication (*n* = 1266) | .11 | **<.001** |  | .03 | .338 |  | .01 | .838 |
| Only MIDUS 2 data (*n* = 1054) | .10 | **.002** |  | .03 | .292 |  | .01 | .738 |
| Only MIDUS Refresher data (*n* = 746) | .07 | **.050** |  | .00 | .890 |  | -.01 | .721 |
| **Adjustment for multiple comparisons** |  |  |  |  |  |  |  |  |
| Adjustment with Hommel procedure | .08 | **.005** |  | .01 | .973 |  | .00 | .973 |
| Adjustment with Bonferroni procedure | .08 | **.005** |  | .01 | 1.00 |  | .00 | 1.00 |
| Adjustment with Benjamini-Hochberg procedure | .08 | **.003** |  | .01 | .675 |  | .00 | .973 |

**Table B**

*Coefficients of Gratitude Predicting LDL Cholesterol Levels in Sensitivity Analyses*

| Variation in analysis | Model 1 | |  | Model 2 | |  | Model 3 | |
| --- | --- | --- | --- | --- | --- | --- | --- | --- |
|  | β | *p* |  | β | *p* |  | β | *p* |
| **Original results (for comparison)** | -.00 | .847 |  | -.02 | .425 |  | -.02 | .460 |
| **Alternative data pre-processing choices** |  |  |  |  |  |  |  |  |
| Gratitude reflected and log-transformed | .01 | .760 |  | .02 | .342 |  | .02 | .348 |
| LDL level log-transformed (after winsorization at 3 *SD*) | -.01 | .709 |  | -.02 | .312 |  | -.02 | .346 |
| Winsorization (3 *SD*) applied to all variables | -.01 | .832 |  | -.02 | .388 |  | -.02 | .403 |
| Winsorization (4 *SD*) applied to all variables | -.00 | .912 |  | -.02 | .483 |  | -.02 | .471 |
| **Sub-sample analyses** |  |  |  |  |  |  |  |  |
| Only participants with complete data (i.e., listwise deletion; *n* = 1704) | -.01 | .670 |  | -.03 | .261 |  | -.03 | .308 |
| Only participants not taking antihyperlipidemic medication (*n* = 1266) | -.02 | .455 |  | -.02 | .543 |  | -.01 | .662 |
| Only MIDUS 2 data (*n* = 1054) | -.02 | .444 |  | -.02 | .477 |  | -.01 | .722 |
| Only MIDUS Refresher data (*n* = 746) | .01 | .838 |  | -.02 | .527 |  | -.02 | .570 |
| **Adjustment for multiple comparisons ^b^** |  |  |  |  |  |  |  |  |
| Adjustment with Hommel procedure | -.00 | .973 |  | -.02 | .973 |  | -.02 | .973 |
| Adjustment with Bonferroni procedure | -.00 | 1.00 |  | -.02 | 1.00 |  | -.02 | 1.00 |
| Adjustment with Benjamini-Hochberg procedure | -.00 | .953 |  | -.02 | .675 |  | -.02 | .675 |

#### Figure A

*Serial Mediation Model for HDL Cholesterol Levels*

Dispositional gratitude

Healthy Eating Index

Body Mass Index

Blood HDL levels

*a*1 = .07 **

*a*2 = -.01

*a*3 = -.07 **

*b*1 = .12 ***

*b*2 = -.29 ***

*c*’ = .00

*c* = .01

#### Figure B

*Serial Mediation Model for LDL Cholesterol Levels*

Dispositional gratitude

Healthy Eating Index

Body Mass Index

Blood LDL levels

*a*1 = .07 **

*a*2 = -.01

*a*3 = -.07 **

*b*1 = -.08 **

*b*2 = .05 *

*c*’ = -.02

*c* = -.02
